# The impact of nurse staffing levels on nursing-sensitive patient outcomes: a multilevel regression approach

**DOI:** 10.1007/s10198-021-01292-2

**Published:** 2021-04-19

**Authors:** Karina Dietermann, Vera Winter, Udo Schneider, Jonas Schreyögg

**Affiliations:** 1 Hamburg Center for Health Economics, University of Hamburg, Esplanade 36, 20354 Hamburg, Germany; 2 Schumpeter School of Business and Economics, University of Wuppertal, Rainer-Gruenter-Str. 21, 42119 Wuppertal, Germany; 3 Health Care Management at Techniker Krankenkasse, Bramfelder Straße 140, 22305 Hamburg, Germany

**Keywords:** Nurse staffing, Nursing-sensitive patient outcomes, Quality of care, Acute care, Multilevel models, J11

## Abstract

The goal of this study is to provide empirical evidence of the impact of nurse staffing levels on seven nursing-sensitive patient outcomes (NSPOs) at the hospital unit level. Combining a very large set of claims data from a German health insurer with mandatory quality reports published by every hospital in Germany, our data set comprises approximately 3.2 million hospital stays in more than 900 hospitals over a period of 5 years. Accounting for the grouping structure of our data (i.e., patients grouped in unit types), we estimate cross-sectional, two-level generalized linear mixed models (GLMMs) with inpatient cases at level 1 and units types (e.g., internal medicine, geriatrics) at level 2. Our regressions yield 32 significant results in the expected direction. We find that differentiating between unit types using a multilevel regression approach and including postdischarge NSPOs adds important insights to our understanding of the relationship between nurse staffing levels and NSPOs. Extending our main model by categorizing inpatient cases according to their clinical complexity, we are able to rule out hidden effects beyond the level of unit types.

**Supplementary Information:**

The online version contains supplementary material available at 10.1007/s10198-021-01292-2.

## Introduction

Many factors affect staffing in hospitals, including changes in patient and population characteristics, levels or mechanisms of reimbursement for hospital services, professional development opportunities or requirements, workplace resources, and the overall demand for hospital care [3, 7, 36]. Reforms in hospital financing, such as the prospective payment systems implemented in many European countries and beyond since the 1980s, have generally increased the financial pressure on hospitals and have led both to initiatives to increase hospital efficiency and to more restrictive staffing policies [24]. In recent years, concerns have been raised in a number of countries about insufficient staffing ratios and their potentially deleterious effects on quality of care, leading to minimum staffing regulations in jurisdictions such as California in the US, Victoria in Australia, and, since early 2019, Germany as a whole [12, 21].

Given the trade-off between efficiency and quality, achieving quality-assuring nurse staffing levels at minimal costs in hospitals requires a profound understanding of their impact on nursing-sensitive patient outcomes (NSPOs). Although many studies have provided evidence of a significant relationship between nurse staffing levels and NSPOs [10, 26], these have been subject to limitations such as small sample sizes or statistical methods that do not deal with endogeneity. Moreover, while previous research has recommended combining patient-level data and information on staffing levels at the hospital unit level to examine this relationship more precisely, few studies have done so to date [6]. A further limitation of most previous studies is their failure to consider NSPOs that take place after discharge from hospital, such as readmissions [11, 45]. Additionally, few studies to date have focused on the German market [4, 30]. This is unfortunate given recent legislation mandating minimum nursing ratios in Germany and the need to support their development and implementation with evidence. While evidence from other countries does exist, its transferability is probably very limited due to differences in nurses’ tasks and competencies from country to country.

To address these research gaps, we conduct a comprehensive empirical analysis of the impact of nurse staffing levels on seven NSPOs with staffing information at the hospital unit level in a large sample of German hospitals. In addition to a set of five inpatient NSPOs, we include two NSPOs that occur post-discharge. We account for the grouping structure of the underlying data set by estimating a two-level generalized linear mixed model (GLMM) with inpatient cases at level 1 and unit types at level 2, and by incorporating risk-adjustments. We define a hospital unit as an operating unit within a hospital that focuses on specific types of patients (e.g., internal medicine, geriatrics or cardiology). Extending our GLMM using additional inpatient-case-related characteristics, we address endogenous effects beyond the level of hospital units. Based on prior literature, we derive expectations on the nursing sensitivity of different NSPOs and hospital unit type categories and compare and discuss our findings with these expectations.

## Previous literature

Internationally, the body of literature on the relationship between nurse staffing levels and NSPOs has grown to the extent that the topic has become the focus of several literature reviews and meta analyses, e.g., [13, 26]. Blume et al. [10] performed an umbrella review covering 15 literature reviews that themselves include 201 primary studies conducted between 1990 and 2017. They found that many studies provide evidence of a systematic relationship between nurse staffing levels and NSPOs, yet they also observed large variation in how sensitive these outcomes are to nursing.

Even though most researchers have found that increased nursing hours improve patient outcomes [10, 26], the topic has remained a focus of study (e.g., [13, 39]) due to its international relevance for policy makers and some unsolved challenges in deriving robust and reliable empirical evidence. With regard to the latter, Cook et al. [9] specify two main endogeneity problems in the research. The first is a particular form of *omitted variable bias* resulting from the fact that variation in nurse staffing dedicated to patient care is influenced by many hospital- and hospital-unit-related dimensions, such as hospital equipment or working conditions. For instance, a higher standard of medical equipment might reduce the required number of nurses for patient care, or vice versa. The second problem refers to *endogenous sorting*, whereby hospitals devote more resources to patients with a higher risk of adverse outcomes, leading to an increase in nurse staffing levels in hospitals or hospital units with more severe inpatient cases. While endogenous sorting can be controlled for through risk adjustment, the effectiveness of such techniques depends on how well observable measures actually depict the true case severity of patients.

One further challenge is the level of data analyzed. The majority of studies to date have examined the association between nurse staffing levels and NSPOs using staffing data at the aggregated hospital level [1, 8, 26]. This may represent a major source of bias because relying on such data makes it difficult to address endogeneity from variation at the hospital unit level. To gain better insight into the relationship between nurse staffing levels and NSPOs, several authors have recommended analyzing this association at the level of hospital units [10, 43]. So far, however, only a few studies have accounted for the level of hospital units, probably because data on staffing at the unit level are not included in publicly available data sets [6]. While some of the unit-level studies have incorporated unit-level information by controlling for fixed effects for hospital units or unit types (e.g., [34, 45]), others have stratified data sets to obtain differences across unit categories (e.g., [14, 33]). Even though these studies have found variation in the associations between nurse staffing levels and NPSOs across hospital units, they have still addressed only some of the endogeneity problem because of their reliance on small sample sizes and their rather coarse distinction between categories of hospital unit (e.g., between only medical and surgical units). To address this limitation, Milstein & Schreyögg [30] conducted a cross-sectional analysis of a large data set covering almost all hospital units in Germany from 2012 to 2014. Based on approximately 27 million inpatient cases stratified across 20 distinct unit types, they used ordinary least squares (OLS) regressions to analyze the link between nurse staffing levels and 11 NSPOs. They observed substantial variation in the number of significant results across the different unit types.

From a methodological perspective, the OLS regression model applied by Milstein & Schreyögg [30] corresponds to the design of the majority of studies in this field, which mainly use linear or logistic regression models [1, 4, 6, 33, 43]. So far, multilevel regression models have been employed only rarely even though they may have advantages in this context. Indeed, such models make it possible to account for different levels of aggregation in data samples (e.g., patients grouped in hospitals, unit types, or both) and to avoid the restriction that estimated coefficients are constant across individual cases [17]. However, of studies that have used multilevel regression to examine the relationship between nurse staffing levels and NSPOs to date [8, 11, 21, 39], only a few have incorporated random slopes for individual unit types [11, 21]—even though research suggests that this relationship differs substantially across unit types [14, 33].

In the present study, we address this limitation by incorporating unit types as an additional level in our statistical model and estimating random slopes, allowing the impact of the staffing variable on our set of NSPOs to vary across individual unit types. Additionally, most studies rely on the inpatient NSPOs proposed by Needleman et al. [33]. However, these vary substantially in their degree of nursing sensitivity [10]. Post-discharge NSPOs (e.g., readmissions) are analyzed in only a few studies, even though these NSPOs might contain additional relevant information related to the hospital stay [11, 45].

To conclude, there is a large amount of evidence for an association between nurse staffing levels and NSPOs. Moreover, the body of literature coming closer to proving causal relationships has grown in recent years (e.g., [34]) but is subject to important limitations. We aim to address some of these limitations in the present analysis. First, by combining claims data from the largest statutory health insurer in Germany with mandatory quality reports published annually by each hospital in Germany, we create a large and rich data set with staffing information at the hospital unit level for our statistical analyses. Second, by adding two post-discharge NSPOs (i.e., 30-day readmissions and 7-day readmissions) to a carefully selected set of inpatient NSPOs, we go beyond the scope of hospital stays and add information that has been neglected by the majority of studies conducted to date. Finally, we address a substantial amount of the endogeneity in studies that have focused on the hospital level. Applying an advanced, two-level GLMM and two different risk adjustments, we more accurately account for the grouping structure of our data set. By including random intercepts and random slopes in our statistical model, we account for variation within and between different unit types and show whether the associations between nurse staffing levels and NSPOs differ among these.

## Methods

### Data and sample

Our study relies on a combination of two data sources: claims data provided by Techniker Krankenkasse, the largest statutory health insurer in Germany covering about 13 percent of all individuals with statutory health insurance in the country, and mandatory quality reports published annually by every hospital in Germany. The claims data contain detailed information on patient-level disease course, allowing us to derive NSPOs that occur either during the hospital stay or post-discharge. We extract all full inpatient stays invoiced in line with the German DRG system between 2014 and 2018 from the data base. We exclude inpatient cases discharged from pediatric, psychiatric, or intensive care units because many NSPOs applied in adult acute care are not applicable to or have not been validated for these hospital units [30, 35]. In addition, we exclude all unit types for which the prevalence rate for each of the NSPOs falls below one percent, as well as unit types that are present in fewer than 25 hospitals.^1^ This leads to 15 different unit types, of which eight unit types predominantly focus on surgical patients (i.e., “general surgery”, “trauma surgery”, “neurosurgery”, “vascular surgery”, “plastic surgery”, “heart surgery”, “urology”, and “dentistry”) and the remaining seven unit types (“internal medicine”, “geriatrics”, “cardiology”, “hematology”, “gastroenterology”, “pneumology”, and “dermatology”) focus on medical patients but might also include surgical patients to varying degrees. Moreover, we remove inpatient cases involving individuals who were not continuously insured in the 90 days before and 90 days after the hospital stay. This leads to an initial sample of 4,589,147 hospital stays in 1358 hospitals.

Quality reports are published annually by each hospital in Germany, and doing so is mandatory. The reports contain general information at the hospital unit level, such as the number of patient cases and staffing numbers. Using the quality reports, we derive data on nurse staffing levels per hospital unit. We remove hospitals with inconsistencies in their reporting.^2^ Moreover, a number of hospitals in Germany belong to hospital groups with only one institutional code but multiple hospital sites. In this situation, we decide on a case-by-case basis whether it is reasonable to combine the individual hospital sites into one hospital group in our analysis based on geographical distance and hospital size.

Following these adjustments, we merge our inpatient cases with the quality report information from the preceding year^3^ using a combination of the institutional code of the hospitals and the department code of the individual unit types, reducing our sample to 3,574,776 inpatient cases in 1147 hospitals. Further details on the sample selection process are shown in Table A.1 of the appendix.

### Nurse staffing

Various ways to measure nurse staffing levels are presented in the literature. These can be summarized into two main measures. The first is the number of nurses working per shift (or over a 24-h period) divided by the number of patients occupying beds over the same period. The second is the number of nursing hours per patient bed days (NHPPD) [13, 41]. The former is frequently used in studies that rely on data from surveys in which nurses provide information on their average patient load per shift (e.g., [1, 2]). In contrast, the latter is more common in studies that rely on administrative data (e.g., [23, 33, 43]).

In our study, we measure nurse staffing in year *t* and hospital unit $u_h$ by computing a patient-to-nurse (PTN) ratio. This measure is an equivalent to the NHPPD ratio over a period of one year and is in concordance with the definition for measuring nursing workload as suggested by the National Office of Statistics [31] in Germany. The PTN ratio indicates how many patients a nurse has to care for during an average shift:

1 $$\begin{aligned} {\text {PTN}}_{t,u_h}=\dfrac{ ( \text {occupation days})_{t,u_h} * 24 \text {hours}}{ (\text {nurses [FTE]})_{t,u_h} * 220 \text {days} * 8 \text {hours}}, \end{aligned}$$

with

2 $$\begin{aligned} \text {(occupation days})_{t,u_h}= (\text {inpatient})_{t,u_h} * (\text {average LoS})_{u_h}, \end{aligned}$$

where the number of nurses (nurses [FTE]) and the number of inpatient cases (inpatient) per year *t* and hospital unit $u_h$ are derived from the quality reports. The total number of nurses comprises all registered nurses (RN) with at least three years of training but also captures a smaller fraction of assistant nurses with at least one year of training.

We derive the average length of stay (LoS) in Eq. (2) from the claims data. However, because the underlying set of claims data covers only a subset (albeit a substantial one) of all hospital patients in Germany, this procedure might induce bias. To mitigate this potential bias, we apply two measures. First, we pooled the length of stay for hospital unit $u_h$ for the years 2014–2018. Although by doing so we reduce variance, this procedure improved model fit and increased robustness of our results compared to a model with repeated cross-sections for each year. It is also reasonable because we do not observe systematic changes in the length of stay for individual hospital-unit combinations $u_h$ across years. Second, we exclude ex ante all hospital units $u_h$ in which the number of inpatient cases over the years 2014–2018 falls below 500.

After obtaining the PTN ratio per hospital unit $u_h$ for each year, we exclude extreme values, i.e., values below one and above 15, reducing our final sample to 3,159,136 inpatient cases in 907 hospitals.

### Nursing-sensitive patient outcomes

We analyse seven NSPOs: five inpatient NSPOs and two post-discharge NSPOs. The choice was guided by the consensus standards for nursing sensitive care for acute hospitals of the National Quality Forum [32] and the results of the umbrella review of Blume et al. [10]. While all outcomes are by definition assumedly sensitive to nurse staffing, they vary in terms of their strength of evidence, i.e., the number of studies analyzing each indicator and detecting significant associations with nurse staffing. In addition, there are slight variations in the underlying assumptions in how far or how strongly nurses causally affect each outcome. For instance, while mortality and readmission are rather general outcomes [19, 26], pressure ulcers and the early detection or prevention of life-threatening complications—including pneumonia and sepsis—are assumed to be in heavy responsibility of nurses and strongly dependent on adequate nurse staffing [8, 10, 26, 27]. Table 1 gives an overview of the NSPOs, their empirical strengths of evidence and assumptions on the causal contributions of nurses.

**Table 1:** Overview of NSPOs

	NSPO	Inpatient/post-discharge	Strength of evidence*	Causal contribution of nurses
(1)	Mortality	Inpatient	Moderate	*Medium*: mortality is a rather general outcome and affected by multiple factors [19, 26]
(2)	Respiratory failure	Inpatient	Moderate	*High*: respiratory failure is part of clinical deterioration and complications, which nurses are more likely to detect if nurse staffing levels are higher [27]
(3)	Pressure ulcers	Inpatient	Low	*High*: frequent position changes provided by adequate staffing can prevent pressure ulcers [8]. Yet, there is also general agreement that detection rates for pressure ulcers are higher if nurse staffing levels are higher [8, 10, 37, 38]
(4)	Pneumonia	Inpatient	Moderate	*High*: pneumonia is part of clinical deterioration and complications, which nurses are more likely to detect if nurse staffing levels are higher [27]; it is part of highly sensitive failure-to-rescue events [26]; nurses are primarily responsible for preventing pneumonia by ensuring adequate respiration among patients at risk, for example by mobilizing them early and having them perform breathing exercises [10]; nurses are heavily responsible for lung care [8]
(5)	Sepsis	Inpatient	Low	*Medium*: sepsis is part of clinical deterioration and complications, which nurses are more likely to detect if nurse staffing levels are higher [27]; it is part of highly sensitive failure-to-rescue events [26], but it might also be highly affected by physicians or only nursing sensitive for selected patients [10]
(6)/(7)	30-d readmission/7-d readmission	Post-discharge	High	*Low/medium*: readmission is a rather general outcome and affected by multiple factors [19, 26], but nurses are often responsible for carrying out discharge preparation functions, which might prevent readmissions [45]. In addition, nurses facing understaffing might be less likely to detect complications or new health problems at early stages. Such complications might become apparent and worsen after discharge, ultimately leading to readmissions [10]; causes of near-term readmissions are much more under the hospital’s control and preventable far more often than later ones [25]

*Strength of evidence based on umbrella review of Blume et al. [10]

We code NSPOs as binary variables (i.e., $=$ 1 if adverse advent occurred). Deaths and readmissions are directly available in the claims data. We derive the remaining NSPOs using the ICD codes for the principal and secondary diagnoses in the claims data. To translate NSPOs into ICD codes, we follow the algorithms developed by Needleman et al. [33]. Because Needleman et al. [33]’s algorithms are based on ICD-9, we rely on McCloskey’s translation of ICD-9 to ICD-10 for New Zealand [29].^4^ We derive inpatient NSPOs based on the whole sample of 3,159,136 inpatient cases. For the post-discharge NSPOs, however, we aim to ensure that the NSPOs are related to a “pure hospital reference stay”—that is, the hospital stay should not represent a readmission itself. Thus, for post-discharge NSPOs, we exclude all inpatient cases from the sample that were preceded 90 days or fewer by another hospital stay. This procedure reduces the number of inpatient cases for the post-discharge NSPOs by almost 30%, from 3,159,136 to 2,279,109.

### Risk adjustment

To account for structural differences in the case severity of inpatient cases across hospitals, we apply two independent comorbidity scores: the Patient Clinical Complexity Level (PCCL), the Elixhauser Comorbidity Measure (ECM)and additionally use age and gender. Patient comorbidities can be used to predict the intensity of resource use and the likelihood of poor outcomes, such as mortality and morbidity. Both, PCCL and ECM are derived from DRG coding information. The PCCL reflects the degree of a patient’s comorbidities and complications and is expressed as an integer ranging from 0 (no clinical complexity) to 6 (highest clinical complexity). It is derived from a closed list of comorbidities and complications and is meant to predict a patient’s need for hospital resources, such as nursing care [18]. For a given patient, PCCL increases with the number of relevant comorbidities and complications. PCCL values are directly available in the underlying claims data used in this study. We directly incorporate the PCCL index in our model because sensitivity analyses using dummy variables for each score suggested a linear relationship. Additionally, we calculate the ECM developed by Elixhauser et al. [15]. The ECM consists of a set of 30 dichotomous comorbidity measures associated with three main outcomes: length of stay, hospital charges, and mortality. In our study, we apply the numeric ECM score developed by van Walraven et al. [44].

### Statistical model

To account for and estimate differences across unit types, we use a multilevel regression model. Such models are advantageous because, by specifying levels of additional effects, they avoid the restriction that coefficients must be constant across individual inpatient cases. In our context, they account for unit types by treating each unit type level as a separate dataset and running separate regressions for each (e.g., [14]). While fixed effects models do not allow the association of interest to vary across unit types [5], fully unpooled models can overstate the variation across unit types and do not account for similarities among them [17]. In contrast, multilevel models include all unit types in a single model but also account for their distinctness [17].

In this study, we estimate a mixed model including random intercepts and random slopes for individual unit types. This is in line with the within-between random effects model (REWB) as proposed by Bell et al. [5], who showed that this model represents the most general model class, covering the strengths of fixed and random effects models, and recommended that it should be used in the context of multilevel modeling. In particular, they argued that including random slopes produces less biased standard errors. Furthermore, in the case of our study, including random slopes is necessary to demonstrate whether the link between the PTN ratio and NSPOs is driven by individual unit types. Because our dependent variables (NSPOs) are dichotomous, we include a link function and perform a cross-sectional two-level GLMM with inpatient cases *i* at level 1 and hospital units *u* at level 2 to estimate the impact of the PTN ratio on our NSPOs. The equation is given by

3 $${\text{NSPO}}_{{iu}}^{{(x)}} = {\text{ }}\alpha _{{0u}} + \alpha _{{1u}} {\text{ }}\widetilde{{{\text{PTN}}_{{iu}} }} + \alpha _{2} {\text{ }}\widetilde{{{\text{PTN}}_{{iu}} ^{2} }} + \gamma X_{{iu}} + \varepsilon _{{iu}} {\text{ }}({\mathbf{level1}}),$$

with

4 $$\alpha _{0u}=\alpha_{0} + \beta \overline{\text {PTN}}_u + {\tilde{u}}_{0u} \quad \text {and} \quad \alpha_{1u}=\alpha _1 + {\tilde{u}}_{1u} \,\,{\mathbf{(level 2) }},$$

where $\text {NSPO}^{(x)}_{iu}=\text {ln}\big (p^{(x)}_{iu}/(1-p^{(x)}_{iu})\big )$ with $\ Y^{(x)}_{iu}|p^{(x)}_{iu} \sim \text {Bern}(p^{(x)}_{iu})$ and $Y_{iu}^{(x)}$ representing the occurrence of an NSPO $x \in \{1,\ldots ,7\}$ for inpatient case *i* admitted to unit type *u*. $\alpha _{0u}$ and $\alpha _{1u}$ represent the random coefficients for the group-varying intercept and slope, allowing for variations across different unit types *u*. $\widetilde{\text {PTN}_{iu}}=(\text {PTN}_{iu}-\overline{\text {PTN}_u})$ corresponds to the mean-centered PTN ratio associated with inpatient case *i* in year *t* and hospital unit $u_h$, and $\overline{\text {PTN}}_u$ with coefficient $\beta$ represents the average PTN ratio per unit type *u*. A squared PTN ratio $\widetilde{\text {PTN}_{iu}}^2$ with coefficient $\alpha _2$ is included in the model specification to allow for non-linear effects. $X_{iu}$ corresponds to a vector of control variables at the first level comprising age, age squared, bed categories (50–299, 300–499, 500–749, and $>750$ beds), year fixed effects, and one of the two risk adjustment procedures PCCL or ECM. $\gamma$ corresponds to the vector of coefficients for $X_{iu}$. $\varepsilon _i, \text {iid} \sim N(0, \sigma )$ is the error at the first level of the regression, while $({\tilde{u}}_{0u},{\tilde{u}}_{1u})$ represents the error at the second level of the hierarchy, following a bivariate normal distribution.

Inserting (4) in (3) and rearranging procedures, we obtain the following composite structure of fixed and random effects:

5 $$\begin{aligned}&\text {NSPO}^{(x)}_{iu}= \nonumber \\&\underbrace{\alpha _0 + \alpha _1 \widetilde{\text {PTN}_{iu}} + \alpha _2 \widetilde{\text {PTN}_{iu}}^2 + \beta \overline{\text {PTN}}_{u} + \gamma X_{iu} }_{\text {fixed effects}} + \nonumber \\&\underbrace{{\tilde{u}}_{0u} + {\tilde{u}}_{1u} \widetilde{\text {PTN}_{iu}}}_{\text {random effects}}+ \varepsilon _{iu}. \end{aligned}$$

Because inpatient cases are grouped not only by unit types *u* but also by hospitals *h*, it would be conceivable to incorporate, additionally, hospitals at the second level of our model. However, computing the intraclass correlation coefficient (ICC) via $\frac{\tau _0^2}{\tau _0^2+\pi ^2/3}$, with $\tau _0^2$ corresponding to the intercept variance, for an empty, unconditional model and a random intercept for either unit types or hospitals [16, 40] suggests that differences in unit types account for a significantly greater amount of the variability in the frequency of the NSPOs. For instance, we find that unit types explain 34.4% of the variation in the mortality rates among inpatient cases, whereas hospitals themselves account for only 11.3% of the variation. Incorporating both groupings (hospital units *u* and hospitals *h*) at the second level of our statistical model results in 11,464 classes at this level, leading to a very complex and inefficient model with a rather low number of observations within individual classes. Therefore, we focus on unit types at the second level of our main analysis and rely on control variables (e.g., bed categories) and risk adjustments to account for differences in hospital characteristics. Furthermore, we do not incorporate an additional panel structure, because we do not observe a significant variation in the PTN ratio between 2014 and 2018.

To test the robustness of our results, we perform several sensitivity analyses. In particular, to reduce the risk of omitted variable bias as a potential source of endogeneity bias we estimate several models with different specifications of risk adjustment measures. First, we re-ran our models with another risk adjustment measure (ECM). While PCCL is a measure of the cumulative effect of a patient’s relevant complications and comorbidities, ECM focusses on general comorbidities. We also ran models including both measures and tested other risk adjustment measures available from the literature.

Second, because previous studies have provided evidence of an interaction between nurse staffing levels and patients’ clinical complexity [11], we further include an interaction term of the PTN ratio with the PCCL risk adjustment.

Third, we complement our GLMM by categorizing inpatient cases according to their clinical complexity (i.e., low: PCCL = 0, medium: PCCL = 1, 2 or 3, high: PCCL$\ge$ 4). The rationale behind this is that prior evidence provides some hints that the severity of patients affect the PTN-NSPO associations [38, 46, 47]. We incorporate additional cluster at level 2 of our GLMM and estimate random slopes for each category in each unit type. In contrast to including an interaction term of the PTN ratio with the PCCL risk adjustment, this approach allows us to rule out hidden effects beyond the level of unit types and to study whether the link between nurse staffing levels and NSPOs per unit type differs in terms of clinical complexity.

Fourth, as suggested by Griffiths et al. [20], we include physician hours per patient day and the ratio of assistant to registered nurses as control variables.

Finally, our readmission models include deceased patients. Although this approach is widely used in the literature, mortality can be regarded as a competing outcome in these models. Therefore, we alternately estimate models in which we eliminate the deceased individuals from the sample and compare this to the conventional estimation.

### Estimation

We use SAS (Release 9.4) for this analysis. We estimate our GLMM using a pseudo likelihood maximization technique (Newton-Raphson Ridge Optimization). For all seven NSPOs $Y_{iu}^{(x)}$, $x \in \{1,\ldots ,7\}$ we assume a Bernoulli distribution and a logit link function.

## Results

### Descriptive results

Our final sample of 3,159,136 inpatient cases comprises patients ranging in age from 18 to 107 years (average: 60.6 years), 42.87% of whom are female. 66.34% of all inpatient cases were admitted to internal medicine (1,258,263) and general surgery (837,653), which represent the largest unit types with the most diverse spectrum of inpatient cases. All other inpatient cases are divided into the remaining 13 unit types.^5^ Table 2 illustrates the distribution of our independent variable, the PTN ratio, across the 15 unit types. While the average PTN ratio over all inpatient cases is 5.84, variation across the unit types is high. The average PTN ratio is lowest in the heart surgery unit, with nurses caring on average for 3.47 patients per shift, and highest in the geriatrics unit, with nurses caring on average for 8.87 patients per shift.

**Table 2:** PTN ratio per unit type

Unit type	Cases	Hospitals	Mean	St.Dev.
Internal medicine	1,258,263	940	6.01	2.03
Geriatrics	25,921	147	8.87	2.08
Cardiology	246,484	163	5.03	2.11
Hematology	46,386	81	5.73	1.74
Gastroenterology	71,595	91	5.94	1.76
Pneumology	46,406	43	5.69	2.13
General surgery	837,653	923	5.68	2.22
Trauma surgery	197,160	286	6.41	2.47
Neurosurgery	55,519	119	5.98	3.33
Vascular surgery	15,993	118	5.37	1.98
Plastic surgery	12,123	54	5.23	2.65
Heart surgery	23,607	60	3.47	1.67
Urology	234,160	336	6.01	2.10
Dermatology	63,617	75	5.57	1.60
Dentistry	24,249	65	5.64	2.39
Total	3,159,136	907	5.85	2.19

The overall prevalence rates range from 0.9% (pneumonia) to 4.5% (respiratory failure) for the inpatient NSPOs, and 9.8% (30-day readmissions), and 2.9% (7-day readmissions) for the three post-discharge NSPOs.^6^ Again, we observe relatively large variation across unit types. For instance, an average of 5.2% of all inpatient cases died during a stay in a hematologic unit compared to 0.1% of all inpatient cases during a stay in a dermatology or dentistry unit.

### Regression results

Table 3 summarizes the findings of our model that uses PCCL risk adjustment. For each NSPO, we present the fixed effects part $\alpha _1$ and the random effects part ${\tilde{u}}_{1u}$ of the random slope $\alpha _{1u}$ that describes the within the effect of the PTN ratio on each NSPO for each unit type *u*. The random effects ${\tilde{u}}_{1u}$ are presented for each unit type *u*. We also present $\alpha _2$ describing the fixed effect of the squared PTN ratio on each NSPO. All coefficients are shown in odds ratios. We highlight in bold whenever a unit-type-NSPO combination reveals a significant random slope $\alpha _{1u}=\alpha _1+{\tilde{u}}_{1u}$ in the expected direction, that is, whenever an increase in the number of patients a nurse must care for during an average shift is associated with a significant higher prevalence of adverse outcomes. A significant random slope either results from a significant fixed effect $\alpha _1$ (combined with a non-significant random effect in the opposite direction), or from a significant random effect ${\tilde{u}}_{1u}$. In other words, the PTN ratio can significantly affect a given NSPO across all inpatient cases *i* independent of the unit type *u* (fixed effect part). In this situation, the effect of the PTN ratio for each unit type then is significant if the unit type-specific random effect does not significantly deviate in the opposite direction. Second, there might be no significant fixed effect, but, for a specific unit type *u*, a significant random effect of the PTN ratio on a given NSPO.

**Table 3:** Impact of PTN ratio on NSPOs (fixed and random effects in odds ratios)

	(1)	(2)	(3)	(4)	(5)	(6)	(7)
Unit type	Mortality	Respiratory failure	Pressure ulcers	Pneumonia	Sepsis	30-day readmissions	7-day readmissions
Fixed effect							
$\widetilde{{PTN}}$ $(\alpha _1)$	0.998	1.049*	1.013	1.026**	1.000	0.994	1.000
	(0.967;1.03)	*(1.014;1.085)*	(0.981;1.047)	*(1.01;1.043)*	(0.982;1.019)	(0.986;1.003)	(0.992;1.008)
$\widetilde{{PTN}}^2$ $(\alpha _2)$	1.000	0.993***	0.999	0.999	1.000	1.000	0.999
	(0.999;1.001)	(0.992;0.994)	(0.998;1.001)	(0.997;1.001)	(0.999;1.001)	(1.000;1.000)	(0.998;1.000)
Random effect ${\tilde{u}}_{1u}$ (per hospital unit type *u*)							
Internal medicine	1.017	**0.976**	1.029	**1.013**	1.011	0.998	0.994
	(0.984;1.05)	**(0.943;1.01)**	(0.995;1.063)	**(0.995;1.031)**	(0.992;1.030)	(0.989;1.007)	(0.985;1.003)
Geriatrics	0.976	0.951$\dag$	0.968	**0.974**	1.001	1.011	1.003
	(0.937;1.016)	(0.913;0.991)	(0.932;1.006)	**(0.949;1.001)**	(0.969;1.035)	(0.991;1.030)	(0.985;1.020)
Cardiology	1.009	**0.992**	1.048*	**1.020**	0.977	1.018***	1.012
	(0.975;1.045)	**(0.958;1.027)**	**(1.010;1.089)**	**(0.996;1.044)**	(0.954;1.001)	**(1.007;1.029)**	(1.000;1.024)
Hematology	1.169***	1.101***	1.153***	**1.025**	0.935***	0.986	0.997
	**(1.125;1.215)**	**(1.057;1.147)**	**(1.101;1.207)**	**(0.994;1.057)**	(0.911;0.960)	(0.968;1.004)	(0.981;1.014)
Gastroenterology	0.999	0.959$\dag$	1.018	**0.981**	0.986	0.992	1.001
	(0.960;1.039)	(0.921;0.999)	(0.974;1.064)	**(0.950;1.013)**	(0.958;1.014)	(0.978;1.007)	(0.986;1.017)
Pneumology	0.991	**1.011**	1.056*	**1.020**	1.036*	0.99	1.000
	(0.951;1.033)	**(0.975;1.049)**	**(1.009;1.105)**	**(0.992;1.048)**	**(1.002;1.071)**	(0.975;1.005)	(0.984;1.017)
General surgery	0.986	**0.966**	0.989	0.969$\dag$ $\dag$	1.003	1.011*	1.008
	(0.954;1.02)	**(0.934;1)**	(0.956;1.023)	(0.95;0.988)	(0.982;1.024)	**(1.002;1.021)**	(0.999;1.018)
Trauma surgery	0.966	0.943$\dag$ $\dag$	0.978	**0.994**	0.987	1.011*	1.002
	(0.929;1.005)	(0.908;0.978)	(0.943;1.014)	**(0.967;1.021)**	(0.957;1.018)	**(1;1.023)**	(0.990;1.015)
Neurosurgery	1.023	**0.992**	1.003	**0.993**	1.017	1.000	0.994
	(0.986;1.06)	**(0.957;1.029)**	(0.963;1.043)	**(0.970;1.017)**	(0.988;1.047)	(0.988;1.012)	(0.980;1.007)
Vascular surgery	0.983	**1.071**	0.947	**1.004**	0.994	0.99	1.001
	(0.902;1.071)	**(0.999;1.149)**	(0.873;1.028)	**(0.961;1.049)**	(0.942;1.049)	(0.968;1.012)	(0.983;1.02)
Plastic surgery	0.942	0.918$\dag$	0.926*	**1.008**	1.002	1.005	0.999
	(0.864;1.027)	(0.853;0.987)	(0.863;0.994)	**(0.966;1.053)**	(0.950;1.056)	(0.984;1.026)	(0.981;1.018)
Heart surgery	0.953*	1.126***	0.947*	**1.003**	1.010	0.994	1.000
	(0.908;0.999)	**(1.084;1.17)**	(0.898;0.997)	**(0.970;1.037)**	(0.974;1.048)	(0.972;1.016)	(0.982;1.018)
Urology	1.010	0.959$\dag$	0.999	**0.987**	1.013	1.008	0.997
	(0.968;1.054)	(0.922;0.998)	(0.961;1.039)	**(0.956;1.019)**	(0.99;1.035)	(0.998;1.019)	(0.985;1.009)
Dermatology	1.018	**1.072**	0.994	**1.012**	1.021	0.979*	0.988
	(0.932;1.112)	**(0.985;1.166)**	(0.93;1.062)	**(0.969;1.057)**	(0.971;1.075)	(0.963;0.996)	(0.971;1.005)
Dentistry	0.977	**0.988**	0.966	**1.001**	1.011	1.008	1.004
	(0.892;1.07)	**(0.937;1.042)**	(0.895;1.043)	**(0.960;1.044)**	(0.958;1.066)	(0.991;1.026)	(0.987;1.021)
Cases	3,159,136	3,159,136	3,159,136	3,159,136	3,159,136	2,279,109	2,279,109

$$*$$ $$p<0.05$$, $$*$$ $$^*$$ $$p<0.01$$, $$*$$ $$*$$ $$*$$ $$p<0.001$$

$\dag$ $p<0.05$,$\dag$ $\dag$ $p<0.01$, $\dag$ $\dag$ $\dag$ $p<0.001$ (if significance of random effect part ${\tilde{u}}_{1u}$ of $\alpha _{1u}$ goes in opposite direction of fixed effect part $\alpha _1$ and is therefore canceled out)

Significant random slopes (in expected direction) highlighted in bold. Significant random slopes occur when the unit type-specific random effect is significant or when there is a significant fixed effect (highlighted in italics), but no significant unit type-specific random effect in the opposite direction

Confidence intervals are given in parentheses

Out of 105 unit type-NSPO combinations, we obtain 32 significant associations in the expected direction yet with large variation across unit types. For three unit types, we observe significant results in the expected direction for four of the NSPOs (cardiology, hematology, and pneumology). For eight unit types, we find significant results in the expected direction for two of the NSPOs (internal medicine, general surgery, trauma surgery, neurosurgery, vascular surgery, heart surgery, dermatology, and dentistry). For the remaining unit types (geriatrics, gastroenterology, plastic surgery, and urology), we only find significant results in the expected direction for one NSPO. While we observe differences in the number of significant associations for medical versus surgical unit types (averages of 2.6 and 1.6, respectively), it seems apparent that this coarse distinction only partially explains inter-unit type variation (standard deviations of 1.3 and 0.5, respectively).

Additionally, we observe differences in the degree of nursing sensitivity between NSPOs. We find that the NSPO pneumonia is significantly associated with nurse staffing levels for 14 unit types, followed by the NSPO respiratory failure (10 unit types); pressure ulcers and 30-day readmissions (three unit types); mortality and sepsis (one unit type); and 7-day readmissions (no unit type). The significant random slopes for the NSPOs respiratory failure and pneumonia are largely driven by the fixed effect part of the random slope, whereas the significant random slopes for all other NSPOs are driven by the random effects parts. The significant fixed effect of the squared PTN ratio on the NSPO respiratory failure, $\alpha _2$, indicates a nonlinear relationship for this NSPO, i.e., that the marginal effect of the PTN ratio decreases with a higher number of patients a nurse has to care for. However, we do not observe this effect for any other NSPO.

While our results show that increases in the PTN ratio are associated with significant increases in the prevalence rates of NSPOs for various unit types, we also obtain five significant random slopes in the opposite direction (i.e., where decreases in the number of patients a nurse has to care for during an average shift are associated with a significantly higher prevalence of adverse outcomes). This result is particularly striking in the heart surgery unit, where two inpatient NSPOs are associated with a significant increase in the prevalence of adverse outcomes as a result of lower nurse staffing levels. Pressure ulcers is the NSPOs that is most frequently significant in the unexpected direction (two unit types), followed by mortality, sepsis, and 30-day readmissions (one unit type).

In this study, we analyze two post-discharge NSPOs (i.e., 30-day readmissions and 7-day readmissions). We do not detect significant associations between nurse staffing levels and the NSPO 7-day readmissions. The NSPO 30-day readmissions, however, further extends the insights from our analysis of inpatient NSPOs. For the cardiology unit, we find that three inpatient NSPOs (respiratory failure, pressure ulcers, and pneumonia) are significantly associated with the PTN ratio in the expected direction. This result is confirmed by the NSPO 30-day readmissions. In addition, we find that 30-day readmissions are significantly associated with nurse staffing levels in unit types where the results of inpatient NSPOs are inconclusive. In the general surgery and trauma surgery units, we observe a significant association only between nurse staffing levels and one inpatient NSPO (i.e., respiratory failure for general surgery and pneumonia for trauma surgery). The significant associations to nurse staffing levels in these unit types are confirmed by the NSPO 30-day readmissions.

Estimating our statistical model while including other risk adjustment specifications did not change our results. Using ECM instead of PCCL, we find that our results remain largely stable. Joint consideration of PCCL and ECM did not improve model fit in most estimations. Other risk adjustment measures were either not feasible, because our patient group is not confined to the older age group (e.g., frailty indexes), or did not increase model fit. Models including PPCL only generally had the best model fit. In addition, including an interaction term of the PTN ratio with the PCCL risk adjustment does not change our main results. Similarly, including physician hours per patient day and the ratio of assistant to registered nurses as control variables does not lead to significant changes in our regression results. Finally, the results for the readmission models with the elimination of the deceased individuals essentially remain the same. The model only slightly loses power due to the lower number of included observations.

### Additional analyses

To help address the further endogeneity of our results, we extend our GLMM approach by categorizing inpatient cases according to their case severity at level 2 of our statistical model. Detailed results of the additional analyses can be found in table A.6 of the appendix. The analysis yields 110 significant random slopes in the expected direction and 19 significant random slopes in the unexpected direction (out of 315 potential unit type—clinical complexity category—NSPO combinations) and increases the proportion of significant results to the total number of results by 6.03% points from 35.24 to 40.95% and the proportion of significant results in the expected direction to the total number of results by 4.44% points from 30.48 to 34.92%. In addition, we find that the number of significant results varies substantially across the clinical complexity categories. For inpatient cases with a low and medium clinical complexity we obtain 39 and 38 significant results in the expected direction and 2 and 5 significant results in the unexpected direction. In contrast, we only obtain 33 significant results in the expected direction, but also 12 significant results in the unexpected direction for inpatient cases with a high clinical complexity, indicating that there might be more endogeneity issues among inpatient cases with a high complexity than among those with a lower clinical complexity.

## Discussion

Our two-level GLMM analysis in a large sample of German hospitals reveals 32 significant relationships between nurse staffing levels and our set of NSPOs in the expected direction for 15 distinct unit types and thus confirms the findings of many previous studies (e.g., [10, 26]). We observe substantial variation in the number of expected significant results across unit types, demonstrating the need of incorporating the unit type level when analyzing the relationship between nurse staffing levels and NSPOs. In line with the suggestions of other researchers, we emphasize the importance of relying on data at the hospital unit level, in particular to avoid neglecting endogeneity of variation from differences in unit types [43].

We find substantial differences in the degree to which individual NSPOs are sensitive to nursing. Among our NSPOs, pneumonia and respiratory failure are those that are most frequently significantly associated with nurse staffing levels in the expected direction. This is in line with our expectations: pneumonia and respiratory failure are classified as highly nursing sensitive failure-to-rescue events, which occur more frequently when nurse resources are insufficient to provide proactive care, cope with the unpredictable, and maintain flexibility [27].

The third NSPO with an assumedly high nursing sensitivity is pressure ulcers: while our three significant results in the expected direction for this NSPO confirm our expectations, the two counterintuitive significant effects undermine the presumption that nurse staffing also relates to higher pressure ulcer detection rates [8, 10, 37, 38]. At the hospital level, these effects might cancel each other out, making significant results unobservable—something that might also be the case for all other NSPOs for which we observe significant results in both directions (i.e., mortality, sepsis, and 30-day readmissions). We find only few significant effects for the more general outcomes mortality and readmission; even though their strength of evidence is moderate or high [10], they can be considered as multi-causal outcomes with only limited impact of nurses. Against our expectations, near-term readmissions do not show more significant associations with NSPOs than 30-day readmissions. There are a number of possible explanations for this difference, including time lags because patients or office-based physicians wait for recovery before readmitting to hospital or the lower frequency of 7-day readmissions.

Potential explanations for the five “counterintuitive” results in the unexpected direction include endogenous sorting as specified by [9] and described as “simultaneity” by [20]; counteracting effects in the sense that a decrease in the number of patients a nurse must care for increases the probability of detecting an inpatient NSPO; and additional omitted variable biases at the patient, hospital, or hospital unit level (e.g., turnovers during hospital stays, the quantity or quality of hospital equipment, hospital management requirements, and integrated care programs) [9, 10, 28]. For instance, the unexpected significant result for the NSPO sepsis in the hematology unit might be explained by the fact that cancer patients are immunosuppressed after chemotherapy which leads to higher risks of fulminate inflammations such as sepsis. However, these patients, due to their high degree of illness, might also be in higher need for nursing care and might therefore be admitted to better-staffed hospital units. In addition, for some unit types, significant results in the unexpected direction might be due to a large fraction of high complexity patients. In these cases, hospitals might dedicate higher staffing levels to these units, but at the same time might face more adverse events due to the high urgency and complexity of these inpatient cases. One example for this might be the heart surgery unit where more than 35% of the patients are in the highest clinical complexity category and where two of seven NSPOs reveal a significant association to nurse staffing levels in the unexpected direction.

Our study also allows to investigate inter-unit type variation. The higher number of significant associations for medical unit types compared to surgical unit types is consistent with previous research [8, 33, 47]. The difference in effects might be explained by surgical patients being healthier (i.e., as a precondition for being eligible for surgery) and therefore being less dependent on nursing care. Yet, we see that this coarse distinction only explains some of the inter-unit type variation. Another potential assumption to explain variation could be that hospitals may only deviate from an accepted norm for unit types where they expect no impact of variation. To get an impression whether this could be the case, we plot the average PTN ratio, the standard deviation of the PTN ratio and the number of significant PTN-NSPO associations for each unit type (see Fig. 1 of the appendix). While observing substantial variation, it becomes apparent that those unit types with the highest number of significant results in the expected direction, i.e., hematology, cardiology, and pneumology, all have below-average variation in nurse staffing levels and below-average PTN ratios. Conversely, this might indicate that hospitals have higher PTN ratios and more variations in PTN ratios in unit types where they expect only a weak impact on the quality of care. In addition, surgical unit types tend to exhibit higher variation. As mentioned above, they seem to be less nursing sensitive, which provides further support that hospitals align more strongly with norms in more nursing sensitive unit types.

Our extended GLMM analyses suggest that changes in NSPOs are multifactorial and confirm the endogeneity concerns of researchers in this field (e.g., [9]). In particular, we find that some of the remaining endogeneity seems be to related to the clinical complexity. Classifying inpatient cases according to their clinical complexity, we find that the proportion of significant results rises which might be explained by the fact that endogenous sorting between the clinical complexity categories might have been ruled out. We observe a slightly higher share of significant effects for low- and medium-complexity patients. This is in line with similar findings of Shuldham et al. [38] and Winter et al. [47]. A potential explanation might be that for high-severity patients, the quality of cooperation and consistency within and across occupational groups might be more decisive than mere nurse staffing levels [42]. Additionally, we find that the majority of significant results in the unexpected direction are present in high complexity patients. It seems reasonable that outcomes of high-complexity patients depend on a high number of factors apart from nurse staffing such that ommitted variable bias might be most present in these category of patients. Additionally, effects of endogenous sorting might be more pronounced in high complexity patients who are more dependent on nurse staffing compared to patients with a lower complexity. Overall, the greater number of significant results in the unexpected direction suggests that there might be insufficient risk adjustment for specific types of inpatient cases such as those with high clinical complexity or admitted to specific unit types, such as the heart surgery unit.

Although our study yields interesting insights, it has several limitations, each of which offers avenues for further research. It is important to emphasize that, although we improve by addressing a number of endogeneity concerns, there are still several sources of potential bias in our study. First, the independent variable of our statistical model, the PTN ratio, might be subject to sampling bias. The number of occupation days in the numerator of the ratio relies on a proxy of the average length of stay in each hospital unit $u_h$ which is derived from the fraction of inpatient cases in our claims data (13% of all individuals with statutory health insurance in Germany). By using data from five years instead of one and removing hospital units with fewer than 500 observations, we can attenuate this bias but not rule it out completely.

Second, due to limitations in data availability we use the average PTN ratio for each hospital unit for one year. Thus, we do not observe potential variation in staffing deployment or patient load during this year. Using this yearly aggregation we most likely introduce some common measurement bias in our explanatory variable [22]. Although PTN ratio is determined on unit level compared to hospital level and, therefore, reduces potential measurement bias, we still have to acknowledge that the yearly aggregation may lead to measurement bias, that should be considered.

Third, our analysis of hospital units is based on the unit from which inpatient cases were discharged. Potential patient turnovers during the hospital stay are not captured in our analysis because this information is not reliably included in our data sample. Finally, even though it addresses a substantial portion of the endogeneity problems in existing research, our extended multilevel analysis including clinical complexity categories categories at level 2 and significant results in the unexpected direction suggest the presence of remaining unexplained effects. It is quite likely that, although we performed several sensitivity checks, our risk-adjustment measure does not capture sufficient case-mix induced variance in some of the hospital units. In particular, our further analyses suggest that omitted variable bias may exist for hospital units with a high proportion of highly complex patients. Excluding those units lead to results that are more parsimonious. To address these limitations, we suggest applying our two-level GLMM to more data samples and countries, including more detailed information such as day-to-day variations in nurse staffing levels, and to investigate counterintuitive results further in future studies, for example by developing additional risk adjustment classifications, improving by capturing highly complex patients, or by including cross-level interaction effects.

## Conclusion

In this study, we provide evidence of the impact of nurse staffing levels on seven NSPOs in German hospitals. Estimating a two-level GLMM with unit types at level 2, we observe significant relationships between nurse staffing levels and NSPOs for several unit types. Most notably, we find that the number of significant results differs substantially across unit types and NSPOs, and that understudied NSPOs such as post-discharge NSPOs add relevant insights to our understanding of the relationships between nurse staffing levels and NSPOs. Additional analyses show that changes in NSPOs also depend on other patient characteristics, such as clinical complexity of inpatient cases. We, therefore, emphasize the importance of relying on homogeneous groups of inpatient cases when studying the link between nurse staffing levels and NSPOs.

Regardless of its limitations, our research has several important strengths compared to previous studies that have analyzed the link between nurse staffing levels and NSPOs. First, we draw upon a large and carefully selected sample of data at the hospital unit level from hospital quality reports and data at the individual level from claims data comprising a final sample of 3,159,136 inpatient cases in 907 distinct hospitals. Second, we go beyond the scope of hospital stays and show that post-discharge NSPOs contain relevant information related to a preceding hospital stay in three hospital units (i.e., cardiology, general surgery, and trauma surgery). Finally, we apply a two-level GLMM in combination with two different risk adjustments to account better for the grouping structure of our data sample. By using this statistical model, we account for variation within and between different unit types. In this way, we address a significant portion of the endogeneity not only in previous studies conducted at the hospital level, but also in studies conducted at the hospital unit level, the latter of which have approached the problem only by stratifying their data or including fixed effects. Extending our GLMM by categorizing inpatient cases according to their clinical complexity, we are able to rule out expected hidden effects beyond the level of unit types. Based on our findings, we do not claim to provide evidence for a causal relationship between nurse staffing levels and NSPOs. However, due to our strong empirical approach and rich underlying data set, we believe that the results of our study come closer to causality compared to the results of many previous observational studies in this field.

Our results have several implications for management and policy. We provide further evidence that there is a link between nurse staffing levels and NSPOs. In particular, we show for Germany that this association varies by unit type. Variation among unit types may be different in other health care systems. This understanding can help hospital managers better allocate nursing resources and might support policy makers in developing measures to ensure adequate staffing levels. In particular, the differences we observe among unit types and clinical complexity categories are relevant for designing minimum staffing regulations, which are currently one of the most common approaches to improving nurse staffing in hospitals.

## Supplementary Information

Below is the link to the electronic supplementary material.

Supplementary file1 (PDF 227 kb)

## Footnotes

1 We exclude the unit types “gynecology”, “obstetrics”, “otorhinolaryngology”, “ophthalmology”, “nuclear medicine” and “orthopedics” due low prevalence rates as well as “nephrology”, “endocrinology”, “rheumatology”, “pulmonary/bronchial medicine”, “thoracic surgery” and “radiotherapy” because these were present in fewer than 25 hospitals in the final sample.

2 For example, if the inpatient and staffing numbers per hospital unit do not add up to the total number reported at the hospital level.

3 For instance, we combine hospital stays ending in 2015 with quality report information from 2014. This procedure is reasonable because the quality reports mature at the end of the year.

4 A list of NSPOs and related ICD-10 codes is shown in Table A.2 of the appendix.

5 Detailed descriptive statistics at the unit type level can be found in Table A.3 of the appendix

6 For each NSPO, prevalence rates per unit type can be found in Table A.4 of the appendix.
