# Supplementary material for: The impact of nurse staffing levels on nursing-sensitive patient outcomes: a multilevel regression approach
Source: Eur J Health Econ. 2021 Apr 19;22(5):833–46. doi: 10.1007/s10198-021-01292-2 (PMC8214586; doi:10.1007/s10198-021-01292-2)
Supplement: Supplementary file 1 — Supplementary file1 (PDF 227 kb) [file 10198_2021_1292_MOESM1_ESM.pdf]

A Appendix

Tables

Table A.1: Sample reduction procedure per hospital unit (2014–2018)

	All hospital stays			After combination with quality reports			After final exclusions (final sample)		
**unit type**	hospitals	hospital units†	inpatient cases	hospitals	hospital units†	inpatient cases	hospitals	hospital units†	inpatient cases
internal medicine	1,152	6,403	1,654,139	1,003	4,504	1,338,560	745	3,554	1,258,263
geriatrics	309	1,608	82,743	240	1,024	62,930	36	176	25,921
cardiology	219	1,148	334,805	176	786	255,312	147	695	246,484
hematology	128	650	85,902	98	411	56,188	54	260	46,386
gastroenterology	122	657	115,653	97	430	80,797	65	318	71,595
pneumology	66	325	67,259	48	210	51,416	28	139	46,406
general surgery	1,144	6,352	1,193,158	975	4,375	931,593	683	3,227	837,653
trauma surgery	413	2,047	307,020	321	1,375	229,381	203	958	197,160
neurosurgery	177	980	96,503	141	652	74,399	68	323	55,519
vascular surgery	205	1,077	70,998	161	686	48,483	19	91	15,993
plastic surgery	103	515	31,451	81	351	25,891	15	71	12,123
heart surgery	74	431	41,323	64	308	34,204	28	137	23,607
urology	526	2,886	356,445	425	1,887	276,493	224	1,073	234,160
dermatology	113	638	107,004	89	417	73,456	55	273	63,617
dentistry	172	838	44,744	121	498	35,673	34	169	24,249
**total**	**1,358**	**26,555**	**4,589,147**	**1,147**	**17,914**	**3,574,776**	**907**	**11,464**	**3,159,136**

Table A.2: Overview NSPOs relying on ICD10-codes

**NSPO**	**ICD10-codes**
**(2) respiratory failure**	**J18.2**: hypostatic pneumonia, unspecified organism<br>**J81**: pulmonary edema<br>**J95.1**: acute pulmonary insufficiency following thoracic surgery<br>**J95.2**: acute pulmonary insufficiency following nonthoracic surgery<br>**J96.0**: respiratory failure, unspecified<br>**J80**: acute respiratory distress syndrome
**(3) pressure ulcers**	**L89**: chronic ulcer of skin
**(4) pneumonia**	**U69.00**: in hospital acquired pneumonia, at least 48h after admission or manifestation $\leq$ 28 days post-discharge (patients >18 years)
**(5) sepsis**	**A40**: streptococcal sepsis<br>**A41**: other sepsis<br>**A49.9**: bacterial infection; unspecified site<br>**R57.2**: septic shock<br>**R65.0**: SIRS of non-infectious origin without acute organ dysfunction<br>**R65.1**: SIRS of non-infectious origin with acute organ dysfunction<br>**R65.2**: SIRS of infectious origin without acute organ dysfunction<br>**R65.3**: SIRS of infectious origin with acute organ dysfunction<br>**R65.9**: SIRS, not further specified

Table A.3: Characteristics of inpatient cases (in percent)

	cases	LoS	age brackets				sex brackets		bed brackets			
**unit type**	#	mean	<50	50–62	63–75	>75	f	m	50–299	300–499	500–749	>750
internal medicine	1,258,263	5.7	20.1	22.9	25.4	31.7	43.8	56.2	30.0	30.0	20.7	19.3
geriatrics	25,921	18.8	0.8	1.7	16.9	80.5	56.0	44.0	39.5	15.9	26.4	18.1
cardiology	246,484	4.8	12.5	23.7	30.3	33.5	34.2	65.8	11.7	22.6	34.2	31.5
hematology	46,386	8.4	17.2	28.0	33.3	21.5	41.1	58.9	3.2	19.7	20.9	56.1
gastroenterology	71,595	5.6	24.6	23.7	24.5	27.2	45.4	54.6	4.0	18.8	36.3	40.9
pneumology	46,406	5.6	15.7	30.4	33.1	20.8	37.6	62.4	45.5	14.0	17.9	22.5
general surgery	837,653	5.6	34.6	27.4	20.9	17.1	47.3	52.7	34.2	31.3	18.7	15.8
trauma surgery	197,160	6.1	32.3	24.9	20.2	22.6	50.4	49.6	13.9	21.7	32.3	32.1
neurosurgery	55,519	8.8	29.8	30.0	22.3	17.9	48.1	51.9	2.2	17.4	18.8	61.5
vascular surgery	15,993	7.6	17.0	28.9	30.9	23.3	44.9	55.1	42.7	32.4	21.6	3.3
plastic surgery	12,123	5.7	44.7	26.9	16.2	12.2	54.3	45.7	12.4	41.2	27.8	18.6
heart surgery	23,607	13.6	10.6	26.4	36.0	27.0	24.3	75.7	23.1	11.1	15.3	50.5
urology	234,160	4.6	22.9	23.8	28.4	24.9	22.8	77.2	11.4	23.4	27.1	38.1
dermatology	63,617	5.0	29.3	22.4	22.7	25.5	45.8	54.2	8.3	1.8	8.4	81.4
dentistry	24,249	4.7	41.5	20.9	18.5	19.1	44.6	55.4	7.9	12.4	18.5	61.1
**total**	**3,159,136**	**6.0**	**24.7**	**24.5**	**24.5**	**26.2**	**42.9**	**57.1**	**25.5**	**27.0**	**22.5**	**25.1**

Table A.4: Prevalence rates NSPOs (in percent)

**unit type**	**cases (inp.)**	**(1) mortality**	**(2) respiratory failure**	**(3) pressure ulcers**	**(4) pneumonia**	**(5) sepsis**	**cases (post.)**	**(6) 30-day readm.**	**(7) 7-day readm.**
internal medicine	1,258,263	2.7	7.0	1.7	1.1	2.5	869,065	11.9	3.7
geriatrics	25,921	5.0	7.1	8.9	4.9	3.0	9,528	14.0	4.6
cardiology	246,484	1.6	5.7	0.7	0.8	1.3	174,148	10.6	3.1
hematology	46,386	5.2	4.9	1.9	2.2	7.0	11,254	34.1	11.1
gastroenterology	71,595	2.5	3.2	1.5	0.9	2.6	46,483	13.9	4.0
pneumology	46,406	2.3	11.6	1.3	2.1	1.4	26,607	14.0	3.5
general surgery	837,653	0.6	2.0	0.9	0.6	0.8	686,004	6.4	2.0
trauma surgery	197,160	0.5	1.4	1.1	0.4	0.3	167,983	5.5	1.9
neurosurgery	55,519	2.0	4.9	1.0	1.7	1.0	37,832	9.1	2.8
vascular surgery	15,993	0.7	2.2	1.1	0.5	0.6	12,331	6.2	1.7
plastic surgery	12,123	0.3	1.0	1.0	0.3	0.3	10,132	5.3	1.0
heart surgery	23,607	3.8	20.0	2.7	2.6	3.1	10,099	9.2	2.9
urology	234,160	0.4	0.9	0.6	0.2	1.9	150,002	14.0	3.3
dermatology	63,617	0.1	0.2	0.4	0.1	0.2	48,628	8.5	1.6
dentistry	24,249	0.1	1.5	0.3	0.2	0.1	19,013	9.0	2.6
**total**	**3,159,136**	**1.7**	**4.5**	**1.3**	**0.9**	**1.8**	**2,279,109**	**9.8**	**2.9**

Table A.5: Remaining fixed effects of regression analysis (odds ratios)

**effect**	**(1) mortality**	**(2) respiratory failure**	**(3) pressure ulcers**	**(4) pneumonia**	**(5) sepsis**	**(6) 30-day readmissions**	**(7) 7-day readmissions**
$\overline{\text{PTN}}$	1.093	0.828	1.332***	1.291	1.110	1.039	1.071
	(0.671;1.779)	(0.498;1.377)	(1.128;1.574)	(0.916;1.820)	(0.706;1.743)	(0.804;1.343)	(0.834;1.375)
age	1.005*	1.013***	0.993*	0.996	1.004*	1.026***	1.008***
	(1.000;1.010)	(1.01;1.016)	(0.988;0.998)	(0.99;1.002)	(1.000;1.007)	(1.024;1.027)	(1.005;1.01)
age^2	1.000***	1.000	1.000***	1.000***	1.000	1.000***	1.000*
	(1.000;1.000)	(1.000;1.000)	(1.000;1.000)	(1.000;1.000)	(1.000;1.000)	1.000;1.000)	(1.000;1.000)
gender	0.924***	0.96***	0.988	0.763***	0.891***	0.946***	0.965***
	(0.907;0.942)	(0.948;0.972)	(0.967;1.009)	(0.743;0.783)	(0.874;0.907)	(0.937;0.955)	(0.950;0.980)
bed category 1	0.926***	1.014	1.302***	0.813***	0.81***	0.840***	0.884***
	(0.901;0.952)	(0.996;1.032)	(1.263;1.343)	(0.783;0.844)	(0.788;0.831)	(0.829;0.852)	(0.863;0.905)
bed category 2	1.044**	1.176***	1.172***	0.86***	0.878***	0.929***	0.962***
	(1.017;1.071)	(1.156;1.196)	(1.138;1.207)	(0.83;0.89)	(0.857;0.9)	(0.918;0.941)	(0.941;0.984)
bed category 3	1.07***	1.143***	1.104***	0.922***	0.896***	0.967***	0.980
	(1.043;1.098)	(1.123;1.162)	(1.071;1.138)	(0.891;0.954)	(0.875;0.919)	(0.954;0.980)	(0.958;1.003)
year 1 (2014)	0.849***	0.628***	0.876***	0.652***	0.757***	1.046***	1.051***
	(0.825;0.875)	(0.616;0.64)	(0.847;0.905)	(0.625;0.679)	(0.735;0.779)	(1.031;1.060)	(1.025;1.077)
year 2 (2015)	0.879***	0.674***	0.893***	0.716***	0.776***	1.005	1.034**
	(0.855;0.904)	(0.662;0.687)	(0.865;0.922)	(0.689;0.744)	(0.755;0.797)	(0.991;1.02)	(1.01;1.06)
year 3 (2016)	0.802***	0.71***	0.764***	0.654***	0.778***	0.982*	0.993
	(0.779;0.824)	(0.697;0.723)	(0.74;0.789)	(0.630;0.679)	(0.758;0.799)	(0.969;0.996)	(0.97;1.018)
year 4 (2017)	0.890***	0.850***	0.863***	0.775***	0.865***	0.995	1.004
	(0.866;0.915)	(0.835;0.865)	(0.836;0.891)	(0.747;0.804)	(0.842;0.888)	(0.981;1.009)	(0.98;1.029)
PCCL	2.600***	2.699***	2.925***	3.318***	2.484***	1.240***	1.252***
	(2.582;2.619)	(2.687;2.710)	(2.900;2.949)	(3.282;3.354)	(2.468;2.500)	(1.236;1.244)	(1.245;1.259)
**cases**	**3,159,136**	**3,159,136**	**3,159,136**	**3,159,136**	**3,159,136**	**2,279,109**	**2,279,109**

*$p < 0.05$, **$p < 0.01$, ***$p < 0.001$

Table A.6: Results of extended statistical model incl. clinical complexity scores (odds ratios)

unit type	clinical compl.	cases	(1) mort.	(2) resp. failure	(3) press. ulcers	(4) pneum.	(5) sepsis	cases	(6) 30-day readm.	(7) 7-day readm.
**fixed effect**										
$\widetilde{\text{PTN}}$ (α_1)	–	3,159,136	1.005	1.035**	1.013	1.019**	1.001	2,279,109	0.997	0.999
$\widetilde{\text{PTN}}^2$ (α_2)	–	3,159,136	1.001	0.994***	1.000	1.000	1.001	2,279,109	1.000	0.999
**random effect** $\tilde{u}_{1u}$ **(per hospital unit type** u**)**										
internal medicine	low	690,398	1.033*	1.010	1.016	1.012	1.035**	540,168	0.997	0.999
internal medicine	medium	479,741	1.008	0.998	1.024*	1.011	1.011	287,864	0.994	0.991
internal medicine	high	88,124	1.004	1.007	1.026*	1.014	0.999	41,033	0.995	0.990
geriatrics	low	5,288	1.091	0.930	1.029	0.996	0.964	2,202	1.032***	1.001
geriatrics	medium	16,114	0.993	1.005	0.952**	0.999	1.009	5,682	0.994	1.003
geriatrics	high	4,519	0.946**	1.015	0.989	0.983	1.008	1,644	0.996	1.000
cardiology	low	156,451	1.052*	1.007	1.005	1.001	1.023	118,231	1.024***	1.005
cardiology	medium	76,156	1.022	1.007	1.051**	1.025	0.990	48,327	1.003	1.009
cardiology	high	13,877	0.942***	1.001	1.016	0.999	0.924***	7,590	0.992	1.010
hematology	low	11,912	1.078	1.025	1.031	1.003	0.990	3,255	1.013	0.997
hematology	medium	24,699	1.087***	1.066***	1.001	1.010	0.893***	5,430	0.96***	0.997
hematology	high	9,775	1.161***	1.14***	1.145***	0.995	0.937***	2,569	0.956***	1.000
gastroenterology	low	36,106	1.003	0.994	0.957	0.996	0.979	27,112	0.996	1.004
gastroenterology	medium	30,004	1.046*	1.073***	0.994	1.007	1.022	17,027	0.995	0.996
gastroenterology	high	5,485	0.920***	0.954††	1.029	0.971	0.936***	2,344	1.008	1.003
pneumology	low	21,553	0.994	0.982	1.002	0.996	1.019	15,150	0.981*	0.998
pneumology	medium	21,195	0.989	1.062***	1.039	1.015	1.061*	10,071	0.999	1.002
pneumology	high	3,658	0.971	1.063***	1.040	1.011	1.002	1,386	1.017	1.000
general surgery	low	605,160	0.995	0.997	1.005	0.996	1.003	527,413	0.998	1.013*
general surgery	medium	186,731	0.988	1.000	0.999	0.983	1.007	133,082	1.026***	0.997
general surgery	high	45,762	0.975	1.001	0.984	0.973††	0.998	25,509	1.059***	1.01
trauma surgery	low	149,918	0.984	0.987	1.024	0.997	1.005	133,756	1.005	1.005
trauma surgery	medium	40,331	0.980	0.983	0.958**	1.005	0.995	29,968	1.02**	1.001
trauma surgery	high	6,911	0.946**	0.970†	0.995	0.995	0.968	4,259	1.03***	0.996
neurosurgery	low	35,101	0.961	0.982	0.996	1.000	1.029	25,943	1.008	0.997
neurosurgery	medium	15,848	1.011	1.016	1.031	1.006	1.061**	9,407	0.999	0.989
neurosurgery	high	4,570	1.031	1.035**	0.986	0.994	0.982	2,482	0.922***	1.005
vascular surgery	low	11,039	1.013	1.013	0.981	1.001	1.013	9,257	0.992	1.003
vascular surgery	medium	3,939	1.027	1.01	0.973	1.001	1.011	2,577	1.011	0.999
vascular surgery	high	1,015	0.951	0.973	1.004	1.003	0.972	497	1.048***	0.999
plastic surgery	low	9,648	1.001	0.998	0.999	0.999	1.002	8,480	0.989	0.999
plastic surgery	medium	2,043	0.970	0.918†	0.988	0.999	0.996	1,439	1.016	1.000
plastic surgery	high	432	0.949	0.923†	0.929*	1.008	1.005	213	1.005	1.000
heart surgery	low	4,858	0.98	0.977	0.997	1.000	1.021	2,509	1.073***	0.999
heart surgery	medium	10,390	1.020	1.052***	0.957	1.007	1.072*	4,489	0.991	1.000
heart surgery	high	8,359	0.921***	1.009	0.950*	0.993	0.971	3,101	0.969*	1.001
urology	low	146,378	1.011	0.988	0.972	1.000	1.002	102,343	1.016**	0.997
urology	medium	78,601	0.988	1.012	0.995	0.995	0.992	43,859	0.991	0.997
urology	high	9,181	1.013	0.975	1.005	0.993	1.039**	3,800	1.012	0.999
dermatology	low	47,791	1.001	1.007	1.010	1.002	0.997	38,376	0.959***	0.996
dermatology	medium	14,825	0.979	1.011	1.001	1.000	0.999	9,737	0.969***	0.988
dermatology	high	1,001	1.044	1.047	0.982	1.007	1.059	515	0.996	1.000
dentistry	low	16,399	1.008	0.984	0.999	1.000	1.010	13,720	1.019*	1.006
dentistry	medium	6,955	0.997	1.008	0.992	0.997	0.992	4,844	0.986	1.000
dentistry	high	895	0.963	0.983	0.969	1.003	1.024	449	1.029	1.000

*$p < 0.05$, **$p < 0.01$, ***$p < 0.001$
†$p < 0.05$, ††$p < 0.01$, †††$p < 0.001$ (if significance of random effect part $\tilde{u}_{1u}$ of α_{1u} goes in opposite direction of fixed effect part α_1 and is therefore canceled out)
Significant random slopes (in expected direction) highlighted in gray. Significant random slopes occur when the unit type-specific random effect is significant or when there is a significant fixed effect (highlighted in lightgray), but no significant unit type-specific random effect in the opposite direction.
Confidence intervals are given in parentheses.

Figures

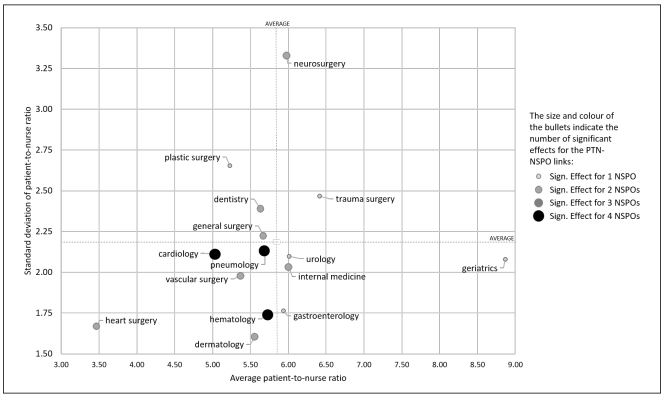

Fig. 2: Effect plot main results
